# Long-term incompatibility of nutritional status and early childhood caries-A call to revamp perinatal and early childhood dietary care and follow-up

**DOI:** 10.3934/publichealth.2023035

**Published:** 2023-06-08

**Authors:** Kavita Sudersanadas, Maha Al Turki, Bahija Basheer, Winnie Philip, Ihssan Hassan Abdelrahman, Alghaliyah Alghofaili, Amani Almoubayed, Abeer Raad Almudaihim

**Affiliations:** 1 College of Applied Medical Sciences, King Saud Bin Abdulaziz University for Health Sciences (KSAU HS), Riyadh, Saudi Arabia; 2 College of Dentistry, King Saud Bin Abdulaziz University for Health Sciences, Riyadh, Saudi Arabia; 3 King Abdullah International Medical Research Centre, Ministry of National Guard Health Affairs, Riyadh, Saudi Arabia

**Keywords:** nutritional status, oral hygiene practices, Dmf scores, BMI, Z scores, preschool children

## Abstract

**Background and aim:**

Dietary pattern and diet quality can influence the incidence of dental caries and can be indicated by decay missing filled (Dmf) scores in the primary dentition. This study aims to find the relationship of nutritional status and oral hygiene practices on Dmf scores of preschool children.

**Materials and methods:**

Semi-structured and self-administered questionnaires, distributed among 60 preschoolers' parents, were used to assess the demographic and diet related data. Anthropometric measurements of the preschoolers were taken following universally accepted protocols. The relationship between dietary supplement consumption and the presence of dental caries was evaluated by chi-square test employing SPSS 22.

**Results:**

The anthropometric measurements among 4-year-old children were on par with the standard values and that of 5-year-olds were below the reference range. All the measurements except head to chest circumference were higher than normal range among 3-year-old subjects. Regular and periodic dental check-ups significantly influenced dental caries prevention (p = 0.030). Statistically, there was no significant association between Dmf scores and thumb-sucking habits (p = 0.568), brushing teeth and usage of tooth floss (p = 0.96), consumption of nutrient supplements (p = 0.744), and BMI (p = 0.564) of the subjects. Furthermore, the correlation between z scores and Dmf scores was found insignificant.

**Conclusion:**

Long-term as well as short-term malnutrition initiated 2 years after the start of the pre-schooling. With severity of undernutrition there was a trend to have high Dmf scores.

## 1. Introduction

Preschool children are at risk of developing malnutrition due to multifactorial etiology. Good nutrition enables children to survive and thrive, whereas malnutrition puts children with adverse consequences [1]. Protein Calorie Malnutrition (PCM), deficiencies of vitamins and minerals like calcium, phosphorus, and iron, can affect oral structures and their development [2]. In addition, these can lead to reduced immunity of the oral cavity against infection and its ability to buffer the plaque acids due to its effect on the functioning of salivary glands [3],[4].

Dental caries leading to demineralization and erosion of enamel has multiple causes [4]. Dental caries begins because of a complicated interaction between acid-producing bacteria and the surrounding environment resulting in the production of lactic and other acids [5]. In addition, the acidic environment in the buccal cavity can also be created due to the fermentation of sugars by *Streptococcus mutans* [6].

Oral health, like general health, depends on the food selected and consumed by a person. Therefore, it is crucial to understand different dietary choices between individuals and their impact on developing dental caries [7]. It was reported that early childhood caries (ECC) is associated with dietary habits [8] and dental hygiene practices [9]. In addition, caries-related microorganisms can be transmitted from parent and/or caregiver to child [10]–[12].

Dental caries was reported as one of the most prevalent non-communicable diseases. The quality of life will be affected by reduced food intake, and there will be changes in the sleeping pattern of persons with dental caries. These may result in pain and chronic systemic infections or adverse growth patterns. Moreover, children with tooth decay are absent from school more frequently [13].

A community's oral health status is usually assessed using Decayed Missing Filled (Dmf) Scores [14]. A healthy eating pattern and diet quality can influence the Dmf scores [15] and, thereby, the incidence of dental caries.

The prevalence of dental caries is lesser among populations with low consumption of fermentable sugars [16]. About 99.1 percent of Saudi Arabian children consumed sugars above the WHO recommendation [17]. Dental caries is highly prevalent among preschool children from Riyadh [18]. Hence, ECC is considered as significant public health issue. The present study investigates the influence of nutritional status and oral hygiene practices on Dmf scores of preschool children from Riyadh.

## 2. Materials and methods

Sixty preschool children attending the preschools of King Saud bin Abdulaziz University for Health Sciences were selected for the study by using a non-random convenience sampling technique. The study followed a cross-sectional study design. The children with metabolic disorders like diabetes, maple syrup urine disease (MSUD), and congenital anomalies were excluded.

A suitably structured questionnaire was used to collect the data related to the demography of the participants. The questionnaire was designed by including demographic data such as nationality, age, gender, family size, and educational and occupational status of the parents, which were assumed to have an impact on quality of life and hence dental hygiene practices and nutritional status.

Body measurements such as height and weight for age and head and chest circumference were measured by trained personnel to assay the children's nutritional status. Data related to the variables affecting the subjects' dental hygiene and feeding practices, such as thumb-sucking habits, brushing teeth, tooth floss, bottle feeding, and bottle feeding while sleeping, were collected from the parents by face-to-face interviews with parents and trained personnel. In addition, the presence of dental caries was documented using the oral health assessment form of WHO and Dmf Scores [19] by a registered dental health care professional.

### 2.1. Data analysis

Frequencies along with percentages were used to denote categorical variables. Mean and Standard Deviation (SD) represent continuous variables. Shapiro-Wilk test was performed to assess the distribution of data.

BMI of the subjects was assessed by using CDC growth charts [20] for children and teens (ages 2 through 19 years).

Z scores for anthropometric measurements were used to assess the child growth and nutritional status relative to a standard or reference population. Z scores were calculated using the formula developed by WHO as $Z=\frac{\left(X-m\right)}{SD}$, in which ‘X’ is the observed values of height, weight or chest circumference, ‘m’ is the mean and ‘SD’ is the Standard deviation of the distribution, corresponding to the reference population [21].

The Z scores for height, weight and head circumference were correlated with the Dmf scores using Pearson Correlation test. The relationship between dental hygiene practice and the presence of dental caries was statistically tested using Pearson's chi-square test. A p value of <0.05 was statistically significant. The data analysis was conducted through SPSS version 22.

### 2.2. Ethics approval of research

The Institutional Review Board of King Abdulla International Medical Research Centre (KAIMRC), National Guard Health Affairs, Riyadh, Saudi Arabia, approved the study protocol. Accordingly, voluntary written informed consent was confiscated from the parents of the study subjects, while oral consent was obtained from the study subjects.

## 3. Results

The demographic distribution of the subjects is shown in Table 1. About 52 % of the subjects belonged to a family of the size of <5 (Table 1). The main proportion (31.7%) of children was the first-born in their families followed by those of second birth order (25%). Twenty-two percent of the subjects belonged to the birth order of ≥5.

**Table 1.** Demographic characteristics of the subjects (n = 60).

Demographic Variable	Number (%) of subjects
Nationality
Saudi	51_(85)_
Non-Saudi	9_(15)_
Age (Years)
3	4_(6.7)_
4	19_(31.6)_
5	37_(61.6)_
Gender
Male	32_(53.3)_
Female	28_(46.7)_
Family Size
<5	31_(52)_
≥5	29_(48)_
Employment status of the parents of the subjects
Employed Father	60_(100)_
Employed Mother	37_(61.7)_
Educational status of the parents	Father	Mother
Up to high school level	12_(20.1)_	12_(20.1)_
Undergraduate/Bachelor	31_(51.67)_	37_(61.7)_
Post-graduation and above	17_(30)_	11_(18.4)_
Total	60_(100)_	60_(100)_

It was found that the level of education of parents (both father and mother) of majority (55.83%) of the participants were of a bachelor's degree or above. Fathers of all subjects were employed; however, 61.7% of the mothers were employed. The family size of the subjects varied between 3 to 9 members.

### 3.1. Nutritional status of the subjects

The subjects' parents informed that most (66.1%) children were not taking any nutrient supplements. Thirty-four percent of them were taking various supplements such as multivitamins (15%), Vitamin D and Calcium (15%), and cod liver oil (1.67%).

**Table 2.** Anthropometric measurements of the participants.

Anthropometric Measurement	Age			
	Mean ± SD			
	3 years	4 years	5 years	Mean of Means
Height for Age (cm)	100.25 ± 1.26	105.48 ± 5.32	109.4 ± 5.53	107.30
*Reference Value 50–85^th^ percentile* [22]	97.50	105.00	111.95	104.80
Weight for age (kg)	16.25 ± 0.96	17.17 ± 2.12	18.10 ± 2.22	17.60
*Reference Value 50–85^th^ percentile* [22]	15.10	17.45	19.60	17.33
Head Circumference (cm)	51.50 ± 1.92	50.41 ± 2.15	50.80 ± 2.60	50.70
*Reference Value 50–85^th^ percentile* [22]	49.75	50.35	51.15	50.41
Chest circumference (CC)	55 ± 4.55	54.78 ± 4.55	56.33 ± 2.57	55.37
*Reference Value for CC* [22]	52.11	53.46	54.55	54.44
Head to chest circumference ratio (HCCR)	0.930	0.920	0.905	0.915
*Reference Value for HCCR* [22]	0.950	0.920	0.910	0.926

The classification of the participants based on BMI showed that the majority (76.67%) were normal, 20% were underweight, and 3.33% were obese. Table 2 indicates the anthropometry of the subjects.

The anthropometric measurements among 4-year-old children were on par with the standard values, and 5-year-olds were below the reference range. On the other hand, all the measurements except head-to-chest circumference were higher than the normal range among 3-year-old subjects.

### 3.2. Dental hygiene practices of the subject

**Figure 1.**
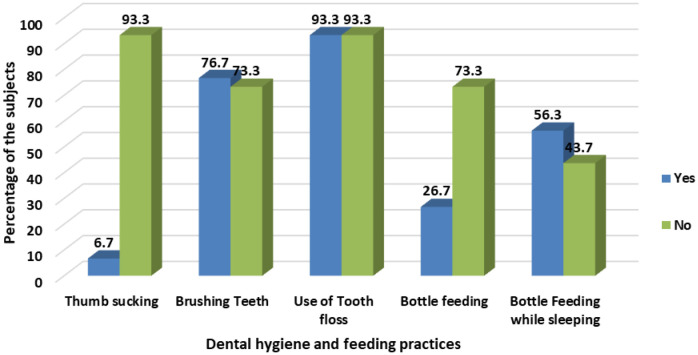
Dental hygiene and feeding practices of the subjects.

The study revealed that most (58.3%) subjects need to have the habit of periodical dental checkups—however, those who visited the dental clinic once a year formed 72 percent of the participants. The subjects' feeding and dental hygiene practices are detailed in Figure 1.

It was noticed that majority of the subjects (73.3%) were not bottle-fed. Among those bottle-fed, 56.3 percent were bottle-fed during their hours of sleep. The habit of thumb-sucking was reported among 6.7% of the children. The majority (76.7%) brushed their teeth regularly, and 6.7% had the practice of using tooth floss.

### 3.3. Dental caries and nutritional status of the subjects

Based on the Dmf scores, dental caries was prevalent among 78.3% of the study subjects. The mean Dmf score was 3.67(±3.63). Among the factors studied, regular and periodic dental check-ups significantly influenced dental caries prevention (p = 0.030). In addition, association between Dmf scores and thumb sucking habits (p = 0.568), brushing teeth and usage of tooth floss (p = 0.96), consumption of nutrient supplements (p = 0.744), and BMI (p = 0.564) of the subjects were insignificant.

**Figure 2.**
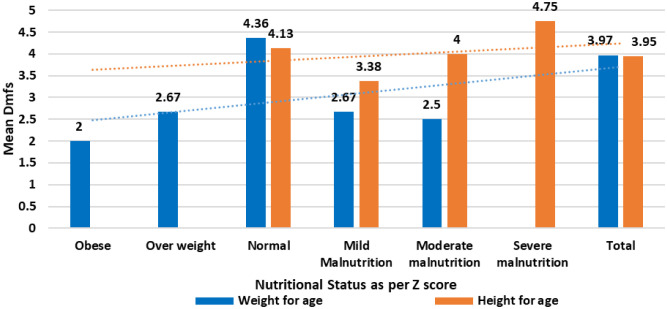
Trend of DMF scores based on nutritional status as per Z score.

Z scores indicated mild to moderate undernutrition among 13.3 percent of the subjects, whereas long-term malnutrition, as evidenced by stunting of different grades, was observed among 36.67 percent. Figure 2 presents the trend of Dmf scores based on nutritional status as per z scores of weight and height for age.

From Figure 2, it was seen that with the advancement in undernutrition, the Dmf scores were also found to increase, especially concerning severe stunting or reduction in height for age. However, the correlation between Dmf scores and the Z scores for height and weight for age, and Head Circumference for the study subjects was found statistically insignificant (Table 3).

It was observed that among 3-year-old children, there was a weak negative correlation for height (−0.377), head circumference (−0.321), and a robust negative correlation for weight (−0.834) with Dmf scores.

**Table 3.** Correlation between Dmf and Z scores for height, weight, and head circumference.

Age	Correlation(r) and p values for Dmf and Z scores					
	Z score for Height		Z score for Weight		Z score for Head Circumference	
	r	p	r	p	r	p
3 years	−0.377	0.623	−0.834	0.166	−0.321	0.679
4 years	−0.024	0.913	−0.081	0.712	0.020	0.929
5 years	0.080	0.658	0.133	0.460	−0.093	0.608

*Note: Significance level at 5%.

For four-year-old children, the correlation between Dmf and Z score for height was weak and negative (−0.024), whereas that for Z scores for weight for age and head circumference was very weak and negative (−0.081) and very weak positive correlation (0.020) respectively. Children of 5 years of age showed a very weak positive correlation for the Z score for height for age (0.080) and weight for age (0.133) and a very weak negative correlation for head circumference (−0.093) and Dmf scores (Table 3).

## 4. Discussion

Few recent studies show the relationship between the prevalence of malnutrition and the incidence of dental caries among Saudi preschool children. Usually, three parameters used for growth monitoring of children include weight and height for age, and the head-to-chest circumference (HC) ratio [23]. The study indicated that as age advances, the incidence of long-term malnutrition arises, as evidenced by the preschool children's low height and weight for age, and head circumference. A study conducted in 2010, indicated that around 13.7% of Saudi children presented with moderate to severe forms of undernutrition [24].

It was observed that the children of three years were above the reference values of age-specific anthropometrics, such as weight for age and height for age. However, they presented with lower head-to-chest circumference than the reference values indicative of protein malnutrition. A low HC measurement is a sign of stunting/growth faltering. It can also predict brain and cognitive development of children during their preschool years [24],[25].

Although 34% of subjects consumed nutrient supplementation, 20% were underweight, and 3.3% were obese. A recent study in Riyadh concluded that 57.9% of the general adult population from Riyadh consumes multivitamin and mineral supplements daily [26].

Our study indicated long-term as well as short-term malnutrition initiated two years after the start of pre-schooling. In addition, the mean anthropometric measurements for 5-year-old subjects were below the reference values indicative of stunting and wasting. This result is on par with the earlier findings that a combination of wasting and stunting was observed in children under five in the central region of KSA [27].

The prevalence of dental caries among the subjects was 78.3%. Among the variables studied, Dmf scores were significantly (p = 0.030) influenced by the periodicity of dental checkups, as reported earlier [28]. The reported data on dental caries prevalence among preschoolers in Riyadh varies between 69–74.8% [28]–[30]. Contradictory to earlier findings [28], our study showed that socio-economic factors did not influence the incidence of ECC.

Various measures to assess the nutritional status and their relationship with Dmf scores were also found statistically insignificant in the study. However, the trend lines indicated that with the severity of undernutrition, Dmf scores showed an increasing trend.

Since 2008, high prevalence rates of ECC have been reported by various researchers [31] with statistically insignificant relation to nutritional status. Hence, hidden maternal malnutrition may be the leading risk factor for the continuous over-reporting of high childhood dental caries among children from Riyadh. Additionally, pregnant women with low vitamin D levels were at risk for developing dental caries in their offspring [31],[32].

## 5. Limitations of the study

The study has certain limitations such as small sample size and fewer study variables. Because of these limitations, we did not control any variable for the analysis.

## 6. Conclusions

Short-term and long-term malnutrition, such as wasting and stunting, along with growth faltering with the progression of age, was observed among preschool children from Riyadh. Hence, they are at risk of adult obesity and slow cognitive development. Irrespective of the socio-economic status, feeding habits, oral hygiene practices, and nutritional status, the prevalence and severity of ECC among preschool children are remarkably high. Based on the present study's results and the earlier research reports, the authors recommend continuous nutritional and dental surveillance and follow-up for pregnant women and preschool and school-going children to reduce the high prevalence rates of dental caries among them. Also piloting a cohort study would have delivered robust evidence on the progressive relationship between nutritional status and dental caries.

## References

[1] United Nations Children's Fund (UNICEF), World Health Organization, International Bank for Reconstruction and Development/The World Bank (2021). Levels and trends in child malnutrition: key findings of the 2021 edition of the joint child malnutrition estimates.

[2] Sheetal A, Hiremath VK, Patil AG (2013). Malnutrition and its oral outcome–a review. J Clin Diagn Res.

[3] Psoter WJ, Reid BC, Katz RV (2005). Malnutrition and dental caries: a review of the literature. Caries Res.

[4] Psoter WJ, Spielman AL, Gebrian B (2008). Effect of childhood malnutrition on salivary flow and pH. Arch Oral Biol.

[5] Pollard TM, Steptoe A, Wardle J (1998). Motives underlying healthy eating: using the Food Choice Questionnaire to explain variation in dietary intake. J Biosoc Sci.

[6] Çolak H, Dülgergil ÇT, Dalli M (2013). Early childhood caries update: A review of causes, diagnoses, and treatments. J Nat Sci Biol Med.

[7] Scardina GA, Messina P (2012). Good oral health and diet. J Biomed Biotechnol.

[8] Davies GN (1998). Early childhood caries—a synopsis. Community Dent Oral Epidemiol.

[9] Berkowitz RJ (2003). Causes, treatment and prevention of early childhood caries: a microbiologic perspective. J Can Dent Assoc.

[10] Davey AL, Rogers AH (1984). Multiple types of the bacterium Streptococcus mutans in the human mouth and their intra-family transmission. Arch Oral Biol.

[11] Berkowitz RJ, Jordan HV (1975). Similarity of bacteriocins of Streptococcus mutans from mother and infant. Arch Oral Biol.

[12] Berkowitz RJ, Jones P (1985). Mouth-to-mouth transmission of the bacterium Streptococcus mutans between mother and child. Arch Oral Biol.

[13] WHO (2017). Sugars and dental caries.

[14] Moradi G, Bolbanabad AM, Moinafshar A (2019). Evaluation of Oral health status based on the decayed, missing and filled teeth (DMFT) index. Iran J Public Health.

[15] Inan-Eroglu E, Ozsin-Ozler C, Ercim R (2017). Is diet quality associated with early childhood caries in preschool children? A descriptive study. Turk J Pediatr.

[16] World Health Organization (2003). Diet, nutrition and the prevention of chronic diseases.

[17] Mumena WA (2021). Consumption of Free Sugar Predicts Nutrient Intake of Saudi Children. Front Nutr.

[18] Al-Meedani LA, Al-Dlaigan YH (2016). Prevalence of dental caries and associated social risk factors among preschool children in Riyadh, Saudi Arabia. Pak J Med Sci.

[19] WHO (2018). Oral Health Surveys: Basic Methods, Ed 5.

[20] CDC (2019). Defining Childhood Obesity | Overweight & Obesity | CDC.

[21] Martinez-Millana A, Hulst JM, Boon M (2018). Optimisation of children z-score calculation based on new statistical techniques. PLoS One.

[22] WHO (2019). Head circumference-for-age.

[23] Sudjarwo SR, Sularyo S, Sudiyanto S (1978). Height and Weight of Preschool children of well-to-do Urban Families in Jakarta City. Paediatr Indones.

[24] El Mouzan MI, Foster PJ, Al Herbish AS (2010). Prevalence of malnutrition in Saudi children: a community-based study. Ann Saudi Med.

[25] Yang Z, Huffman SL (2013). Nutrition in pregnancy and early childhood and associations with obesity in developing countries. Matern Child Nutr.

[26] Musaiger AO, Al-Hazzaa HM, Takruri HR (2012). Change in nutrition and lifestyle in the Eastern Mediterranean Region: Health impact. J Nutr Metab.

[27] Alshammari E, Suneetha E, Adnan M (2017). Growth profile and its association with nutrient intake and dietary patterns among children and adolescents in Hail region of Saudi Arabia. Biomed Res Int.

[28] Alwalan SI, Alrasheed AA, Aldossari KK (2022). Prevalence and characteristics of multivitamin-multimineral (MVMM) use among Saudi populations in Riyadh, Saudi Arabia: A cross-sectional study. Medicine.

[29] AlMarshad LK, Wyne AH, AlJobair AM (2021). Early childhood caries prevalence and associated risk factors among Saudi preschool children in Riyadh. Saudi Dent J.

[30] Al-Meedani LA, Al-Dlaigan YH (2016). Prevalence of dental caries and associated social risk factors among preschool children in Riyadh, Saudi Arabia. Pak J Med Sci.

[31] Wyne AH (2008). Caries prevalence, severity, and pattern in preschool children. J Contemp Dent Pract.

[32] Suárez-Calleja C, Aza-Morera J, Iglesias-Cabo T (2021). Vitamin D, pregnancy and caries in children in the INMA-Asturias birth cohort. BMC Pediatr.

